# Are Cuckoos Maximizing Egg Mimicry by Selecting Host Individuals with Better Matching Egg Phenotypes?

**DOI:** 10.1371/journal.pone.0031704

**Published:** 2012-02-23

**Authors:** Anton Antonov, Bård G. Stokke, Frode Fossøy, Peter S. Ranke, Wei Liang, Canchao Yang, Arne Moksnes, Jacqui Shykoff, Eivin Røskaft

**Affiliations:** 1 Department of Biology, Norwegian University of Science and Technology (NTNU), Trondheim, Norway; 2 College of Life Sciences, Hainan Normal University, Haikou, People's Republic of China; 3 Laboratoire Ecologie, Systematique et Evolution UMR 8079 CNRS-Université Paris-Sud XI-AgroParisTech, Orsay, France; University of Pretoria, South Africa

## Abstract

**Background:**

Avian brood parasites and their hosts are involved in complex offence-defense coevolutionary arms races. The most common pair of reciprocal adaptations in these systems is egg discrimination by hosts and egg mimicry by parasites. As mimicry improves, more advanced host adaptations evolve such as decreased intra- and increased interclutch variation in egg appearance to facilitate detection of parasitic eggs. As interclutch variation increases, parasites able to choose hosts matching best their own egg phenotype should be selected, but this requires that parasites know their own egg phenotype and select host nests correspondingly.

**Methodology/Principal Findings:**

We compared egg mimicry of common cuckoo *Cuculus canorus* eggs in naturally parasitized marsh warbler *Acrocephalus palustris* nests and their nearest unparasitized conspecific neighbors having similar laying dates and nest-site characteristics. Modeling of avian vision and image analyses revealed no evidence that cuckoos parasitize nests where their eggs better match the host eggs. Cuckoo eggs were as good mimics, in terms of background and spot color, background luminance, spotting pattern and egg size, of host eggs in the nests actually exploited as those in the neighboring unparasitized nests.

**Conclusions/Significance:**

We reviewed the evidence for brood parasites selecting better-matching host egg phenotypes from several relevant studies and argue that such selection probably cannot exist in host-parasite systems where host interclutch variation is continuous and overall low or moderate. To date there is also no evidence that parasites prefer certain egg phenotypes in systems where it should be most advantageous, i.e., when both hosts and parasites lay polymorphic eggs. Hence, the existence of an ability to select host nests to maximize mimicry by brood parasites appears unlikely, but this possibility should be further explored in cuckoo-host systems where the host has evolved discrete egg phenotypes.

## Introduction

Brood parasitic birds such as cuckoos and their hosts are involved in complicated coevolutionary arms races [1]. Such interactions lead to evolution of antiparasite defenses by the hosts followed by counter-defenses by the parasite [2], [3]. The most common pair of reciprocal adaptations in host-brood parasite systems is foreign egg discrimination of the hosts and egg mimicry of their parasites [4]. Once parasites evolve eggs that mimic the average host egg phenotype, hosts are selected for more advanced antiparasite adaptations such as decreased intra- and increased inter-clutch variation of egg appearance to facilitate the detection of the parasitic egg [5], [6], [7]. As host interclutch variation becomes more extreme, it may be manifested in egg polymorphism, i.e. dramatically different discrete egg morphs [8]. Host egg polymorphism is a major challenge to brood parasites especially if host egg morphs have similar frequencies. Thus, for the parasite to persist with such a host, it should also evolve corresponding polymorphism. Theory shows that if there is matrilineal inheritance of parasite egg phenotypes and mimicry-dependent egg discrimination by the host, stable egg polymorphism in both parties may evolve [9]. This phenomenon occurs in nature in at least two host-parasite systems in which parasites have discrete egg morphs that match corresponding egg morphs of a single polymorphic host species [10], [11].

Once matching host and parasite egg polymorphism has arisen, particularly when hosts that are able to recognize and reject mismatched parasitic eggs, parasites that preferentially victimize host nests of the right host phenotype would have an advantage. Thus, appropriate host choice by the parasite would be adaptive [12], [13]. However, appealing though this is as a hypothesis, there is no evidence for selection of hosts by parasites in relation to egg morph in these dual polymorphic systems [10], [11]. Nonetheless, brood parasites do not lay in all host nests they have discovered but may prefer some host individuals over others based on host quality and/or behavior [14], [15], [16], [17]. Might brood parasites choose their host nests based on the phenotypic match between their own and the host's eggs when egg phenotypic variation is gradual rather than expressing distinct morphs? This hypothesis has been tested only twice in the common cuckoo *Cuculus canorus* and its reed warbler *Acrocephalus scirpaceus* and great reed warbler *A. arundinaceus* hosts [13], [18], both species having continuous interclutch variation in egg appearance. These studies found that cuckoo eggs in naturally parasitized nests matched their host clutches in some color components better than did unparasitized or experimentally parasitized host clutches.

Here we tested cuckoo-host selection hypothesis in a population of marsh warblers *A. palustris* parasitized by the common cuckoo. We compared egg phenotype matching of host and parasite eggs at parasitized nests and their nearest unparasitized conspecific neighbors using visual modeling and image-processing approaches, and also accounted for host nest availability to cuckoos. This system is relevant for addressing cuckoo-host selection hypothesis because: (1): marsh warblers in the study area are regularly and frequently parasitized by cuckoos [19]; (2) the local marsh warbler cuckoo gens shows specific adaptations to marsh warbler eggs, laying highly mimetic eggs [20]; (3) marsh warblers exhibit refined egg discrimination abilities [19]; (4) hosts have substantial, albeit continuous, interclutch variation [20], creating ample variation in cuckoo egg matching among the different host clutches. We predicted that, if cuckoos discriminate among host clutches, cuckoo eggs should be a better match to the host eggs in the nest where they were laid than in the nearest unparasitized nest principally available for parasitism. In contrast to the few previous similar studies we did not find evidence for cuckoos selecting host nests based on egg matching and argue that such a matching is hardly possible with host species having continuous interclutch variation in egg appearance.

## Results

The mean inter-nest distance was 60.9±33.3 m (range: 14–127). Thus, we assume that nests parasitized by the cuckoo and their nearest unparasitized neighbors were close enough to be detected by the same cuckoo while also distant enough for the cuckoo to perceive them as different nesting attempts. Parasitized nests and their nearest unparasitized neighbors also had similar laying dates (Table 1), differing by a median of 1.5 days (IQR: 0.25–2.50). Parasitized and unparasitized nests also did not differ significantly in the distance to the nearest tree, height of the nearest tree as well as vegetation cover above the nest (Table 1). All the three variables were important correlates in discriminating between parasitized and unparasitized marsh warbler nests in the same population [21]. Thus we can consider that the two nests within a pair were similarly available to parasitism by cuckoos.

**Table 1:** Nest site characteristics and cuckoo-host egg phenotype contrasts at marsh warbler nests naturally parasitised by the common cuckoo and the nearest unparasitised nests.

Variable	Parasitized	Unparasitized	Statistic	df	P
Laying dates(1 = 1 May)	21.36±5.71(10–32)	22.68±4.54(16–35)	1.7613	21	0.09
Distance to nearest tree, m	12.04±5.63(4.5–22.0)	13.89±7.87(4.0–30.0)	149.5 (V)	21	0.46
Height of the nearest tree, m	5.89±3.84(3.0–16.0)	7.25±4.68(3.1–16.0)	101.5 (V)	21	0.09
Vegetation height above nest, cm	115.95±46.43(45.0–200.0)	105.82±54.13(37.0–230.0)	89.5 (V)	21	0.24
Background color, JND	3.258±2.682(0.455–11.770)	3.052±1.623(0.490–6.584)	118.0 (V)	21	0.80
Spot color, JND	5.011±2.609(0.950–11.287)	6.042±3.223(0.805–11.863)	1.22 (V)	21	0.24
Background luminance, JND	2.903±2.168(0.000–8.682)	4.630±4.205(0.424–15.998)	165.0 (V)	21	0.22
Spot cover	0.139±0.076(0.017–0.268)	0.170±0.103(0.005–0.370)	1.47	20	0.16
Spot distribution	0.105±0.067(0.008–0.213)	0.126±0.066(0.010–0.263)	1.00	20	0.33
Spot size, mm^2^	0.173±0.137(0.010–0.488)	0.227±0.341(0.009–1.558)	0.70	20	0.49
Egg volume, cm^3^	1.005±0.203(0.627–1.398)	0.998±0.187(0.695–1.306)	0.18	20	0.86

Data are presented as Means ± SD (ranges). Spot cover and spot distribution are expressed as proportions (see also Material and Methods). Test statistic refers to paired t-tests or Wilcoxon's rank sum tests (indicated as V in brackets), depending on the distribution of the variables. Background and spot color color contrasts as well as luminance contrasts are calculated based on the Vorobyev & Osorio's (1998) perceptual model and the units are JND, meaning “just noticeable differences”. Degrees of freedom are 20 in some cases because for one pair of clutches we did not measure some of the variables.

Cuckoo egg mimicry was not better in parasitized than unparasitized nests with respect to any of the egg characteristics measured, i.e. background and spot color contrasts, background luminance, spotting pattern and egg size (Table 1). Nevertheless, spot cover and background luminance tended to be more closely matched at parasitized than in non-parasitized nests, though the differences were not significant (Table 1).

## Discussion

We found no evidence that cuckoos parasitizing marsh warblers preferentially select host nests based on egg appearance so that mimicry is maximized. Cuckoo egg phenotype match did not differ significantly between parasitized and unparasitized but similarly available nests in spot and background color, background luminance, spotting pattern and egg size. This contrasts with studies on two other *Acrocephalus* warblers, the reed warbler and the great reed warbler, which found some indication that cuckoos may select host nests where mimicry in some color components is higher [13], [18]. This discrepancy in the findings may stem from the different approaches used both in phenotypic measures and in experimental design. Both previous studies used principal components to analyze egg color, while we used a visual modeling technique which, unlike principal component analysis, weights spectra according to host cone sensitivity functions, thereby only focusing on the relevant visual information perceivable by the receiver. Due to sample size limitations, we cannot repeat our analyses using principal components (PCA) to directly compare our results with the aforementioned studies. However, such a step would be of little use anyway since the principal components generated by an analysis are too dependent upon the data entered into the PCA, so PCA scores (and also perhaps differences in scores) from one study cannot be compared directly with those of another study without a re-analysis of the raw data [22], [23]. While principal components can be useful in describing spectral shapes, their use for statistical comparisons of color data has been strongly discouraged for several reasons [24]. Furthermore, unlike the two other studies testing the same hypothesis, we also analyzed background and spot color separately which should be more accurate than simply using random samples over the egg surface which does not control for the differential inclusion of portions of background or spots across measurements. More importantly, these studies took no account of availability of unparasitized nests to cuckoos in terms of their nest-site characteristics and timing. In addition, host selection by cuckoos should be investigated for individual cuckoo females but neither of above studies employed a paired-design in comparing mimicry. [18] compared the phenotype of a few cuckoo eggs to the appearance of reed warbler host eggs in parasitized and unparasitized nests, without directly addressing mimicry within nests. [13] compared egg mimicry in naturally parasitized nests with mimicry in unparasitized nests to which a *different* set of real cuckoo eggs were introduced by the experimenters. Despite these flaws, we cannot dismiss the significant results found by these two studies. Further research using unified approaches and more host species/populations should provide a better answer to this fundamental question of avian host-brood parasite coevolution.

We find the lack of selective phenotypic egg matching by cuckoo females unsurprising for host species with continuously distributed egg phenotypes. Marsh warbler eggs as well as great reed warbler ones show substantial but continuous interclutch variation so that no clear egg morphs can be distinguished [20]. Both species show substantial overlap of host and parasite egg phenotypes in the same Bulgarian population [20], indicative of advanced stages in the coevolutionary arms race. If cuckoos are to choose among nests of such hosts, a perfect knowledge of their own eggs is required and the mechanism behind must involve a memory template as well as remembering egg matching among the different host nests. Cuckoo females must either lay their first egg in isolation or have the ability to distinguish between their own egg and the host eggs already in the nest. Since host species differ substantially in their rejection rates of non-mimetic eggs [1], [25], the benefits of cuckoos matching their eggs within hosts will differ dramatically among cuckoo gentes. Furthermore, in hosts with continuously distributed egg phenotypes, selection would still favor cuckoos mimicking the average host phenotypes [26]. If cuckoos practice such a fine-grained selection of host individuals, this should also enhance a pattern of local adaptation of parasites to their sympatric host egg phenotypes. A recent study on cuckoo and reed warbler egg appearance across Europe showed substantial inter-population differences in host and parasite egg phenotypes but no evidence for local adaptation [27]. Finally, even in host-parasite systems with matching egg polymorphism, where selection by cuckoos of host egg phenotype would be extremely advantageous, there is no evidence that it has appeared. In cuckoo finches *Anomalospiza imberbis* parasitizing tawny-flanked prinias *Prinia subflava* and common cuckoos parasitizing ashy-throated parrotbills *Paradoxornis alphonsianus*, there is dramatic matching egg polymorphism in host and parasite [10], [11] and hosts reject both eggs of the wrong morph and experimentally generated fine-scaled intermediates [11]. Nevertheless, even in these systems there is no evidence that parasites choose the ‘right’ host egg morph [10], [11], even though this possibility requires further investigations. In addition, a theoretical model has shown that egg polymorphism in both hosts and parasites may be stable even with random laying by the parasite [9]. Although an appealing idea, it remains to be shown that selective choice of particular host clutches based on egg phenotype matching by brood parasites would be advantageous and could evolve. Clearly this is not supported by any compelling evidence to date.

Nonetheless, brood parasites exploit their pool of available host nests non-randomly [14], [28]. We paired parasitized nests with their nearest unparasitized neighbors so as to reduce the variation in laying dates and nest site characteristics likely to constrain availability of nests to cuckoos. The two groups did not differ significantly in any of these variables, suggesting that cuckoos could have chosen to parasitise either. Therefore the very existence of these unparasitized nests begs an explanation if cuckoos are, as we think, nest limited. It may be that either: 1) cuckoos failed to find these nests anyway due to factors we did not take into account, e.g. more secretive host behavior [16], or 2) cuckoos knew all the nests but avoided some of them due to host characteristics other than the degree of egg similarity to the parasite's eggs, e.g. host quality [17], [29]. Further studies involving close tracking of individual parasite females and considering a wider range of host characteristics are needed to directly control for actual availability of host nests.

## Materials and Methods

Fieldwork was carried out during 2007–2010 between the villages of Zlatia (43°46′N23°30′E), Ignatovo (43°46′N23°28′E) and Dolni Tsibar (43°48′N23°31′E), north-western Bulgaria. Marsh warblers breed at high densities in diverse but typically reed-dominated vegetation. For more details on the study area see [20].

All the patches of suitable breeding habitat with singing marsh warblers were carefully searched for nests between 15 May and 10 June each year. For the purposes of this study we only considered areas containing at least two neighboring territories adjacent to trees, i.e. readily accessible to cuckoos [21]. To enable correct assignment of the parasitism status, only nests found during the nest building or early egg laying stage were included here. These nests were monitored daily until 5–6 days following clutch completion. Since cuckoos remove 1–2 eggs at laying [3], a would-be fast, hence undetected ejection would be manifest as gaps in the host laying cycle. Furthermore, during the process of puncture ejection, host eggs often become smeared with yolk, a strong indicator that the nest had received a parasitic egg that was ejected [30]. None of the nests classified as “unparasitized” in this study had such indications of undetected parasitism. To further make sure that only nests available to cuckoos were included in the analyses we restricted the sample to parasitized nests and their nearest unparasitized neighbor nest having a similar laying date and positioning, thus creating a paired design (Table 1). Twenty-two pairs of nests passing these rigorous criteria were available for analyses.

A previous study on the same population identified three main nest-site variables discriminating between parasitized and non-parasitized marsh warbler nests: parasitized nests had shorter vegetation covering them from above, were situated closer to trees, and these trees were taller compared to those close to unparasitized nests [21]. Therefore, we measured these characteristics for all the nests in this study. According to our unpublished data, marsh warblers have low intraclutch variation, thus we measured spectral reflectance and size of a randomly selected host egg and the cuckoo egg (if the nest was parasitized) from each nest. Using only one host egg in the context of this study is also justified by the fact that cuckoos typically parasitize host nests before clutch completion [3], [31], [32]. Thus, cuckoos are unable to assess variation in color of the entire clutch before deciding in which nest to lay. Cuckoos also remove 1–2 eggs before laying its own eggs [3], making it impossible for us to measure reflectance of all host eggs within a parasitized clutch. Egg dimensions were taken with a digital caliper to the nearest 0.01 mm and were used to calculate egg volume (cm^3^) as 0.51×length×breadth^2^×1000^−1^ [33]. We measured egg reflectance under standard light conditions by using a USB2000 spectrophotometer with a deuterium halogen light source (D2-W, mini). Each measurement covered ca. 1 mm^2^ and was taken at a 45° angle to the egg surface, with the spectrophotometer and the light source connected with a coaxial reflectance probe (QR-400-7-UV-vis). The spectra were loaded into OOIBASE32 software (Ocean Optics) and interpolated with a step of 1 nm in the range 300–700 nm. We measured reflectance of background color and spots separately because cuckoo and host eggs may differ in the degree of match in these two components of egg coloration [20]. A total of two background and 3–4 spot measures were obtained for each egg. Since in some cases it was difficult to avoid tiny and dense spots for background measurements, we selected the background spectrum with the highest reflectance to represent background color of the egg while spot spectra were averaged. Since human and avian vision differ dramatically [34], we analyzed egg color and luminance by using a perceptual visual model [35]. This model has successfully described thresholds for visual discrimination in birds [35], [36] and also predicted egg rejection behaviour under photopic conditions [37], [38]. By taking into account cone sensitivities, photoreceptor noise and irradiance, Vorobyev & Osorio's (1998) model produces chromatic (ΔS) and achromatic (ΔQ) contrasts between any two stimuli (eggs in our case) in the receptor space. Details about calculations of these contrasts can be found elsewhere [35], [39]. The units for ΔS and ΔQ are JNDs (just noticeable differences). Following [40], we considered discriminabilities below 1 JND to be undetectable by birds, and those with values below 3 JND difficult to distinguish even under favourable light conditions. Recent evidence suggests that there are negligible differences among model calculations obtained using spectral sensitivity data for different passerine species [10]. Thus, given that sensitivity data for cuckoos are not available, we used here single- and double-cone photoreceptor spectral sensitivities, photoreceptor noise, and the transmission properties of ocular media for the blue tit *Cyanistes caeruleus* as representative of UVS type avian visual system which is well understood [41]. Blue tit cone proportions are 1∶1.92∶2.68∶2.7 for SWS1/SWS2/MWS/LWS cones, respectively [41]. Irradiance spectra at the nests of a typical open nester as the marsh warbler was kindly provided by J. M. Avilés based on [42]. We assumed that the signalling noise for each cone e_i_ is independent of light intensity and set the Weber's fraction at 0.05 for all single cones.

We also quantified spotting pattern of cuckoo and host eggs based on photographs of the eggs taken in a standard way on a Kodak Grey plate. Each egg image was divided into three equal sections along the long axis – sharp, middle and blunt. Egg images were processed in Adobe Photoshop CS and IAN software (http://landscape.forest.wisc.edu/projects/ian/) to calculate mean spot cover, spot distribution (proportion of the total spot cover in the blunt egg sector) and spot size. For more methodological details see [20], [38].

Color and luminance contrasts, absolute differences in the three spotting pattern variables and egg volume were calculated for each cuckoo egg in relation to host eggs of its ‘home’ nest and the nearest unparasitized nest. Visual modeling was performed in Avicol software v5 [43]. Contrasts between the two groups were compared via paired t-tests or Wilcoxon's rank sum tests, for normally and non-normally distributed variables, respectively. Statistical tests were run in R.2.11.1 (www.R-project.org).
